# Thermo-Mechanical Fatigue Crack Growth of RR1000

**DOI:** 10.3390/ma10010034

**Published:** 2017-01-04

**Authors:** Christopher John Pretty, Mark Thomas Whitaker, Steve John Williams

**Affiliations:** 1 Rolls-Royce plc, Compressor Rotor Facility, Annesley NG15 0DR, UK; Chris.Pretty@Rolls-Royce.com; 2 Institute of Structural Materials, Swansea University, Swansea SA1 8EN, UK; 3 Rolls-Royce plc, Elton Road, Derby DE24 8BJ, UK; Steve.Williams@Rolls-Royce.com

**Keywords:** TMF, crack growth, RR1000, induction coil, potential drop

## Abstract

Non-isothermal conditions during flight cycles have long led to the requirement for thermo-mechanical fatigue (TMF) evaluation of aerospace materials. However, the increased temperatures within the gas turbine engine have meant that the requirements for TMF testing now extend to disc alloys along with blade materials. As such, fatigue crack growth rates are required to be evaluated under non-isothermal conditions along with the development of a detailed understanding of related failure mechanisms. In the current work, a TMF crack growth testing method has been developed utilising induction heating and direct current potential drop techniques for polycrystalline nickel-based superalloys, such as RR1000. Results have shown that in-phase (IP) testing produces accelerated crack growth rates compared with out-of-phase (OOP) due to increased temperature at peak stress and therefore increased time dependent crack growth. The ordering of the crack growth rates is supported by detailed fractographic analysis which shows intergranular crack growth in IP test specimens, and transgranular crack growth in 90° OOP and 180° OOP tests. Isothermal tests have also been carried out for comparison of crack growth rates at the point of peak stress in the TMF cycles.

## 1. Introduction

Disc materials for high temperature applications in aero engines are predominantly nickel-based superalloys, whose thermo-mechanical fatigue (TMF) lives have been investigated thoroughly by numerous authors, mainly through strain control tests. TMF testing is best explained in terms of phase angles and loading directions. Since there are now two control variables, the phasing between thermal and mechanical cycles usually provides an appropriate description of the TMF condition. Typical cycles usually involve phasing where the stress and temperature increase/decrease concurrently, and the cycle is considered in-phase (IP), or where the stress begins to decrease/increase, the temperature increases/decreases, and the cycle is 180° out-of-phase (OOP). Clearly an infinite number of phase angles between these two extremes are also possible.

Marchionni et al. [1] carried out such work on Nimonic 90 between 400 and 850 °C, finding that OOP produced longer fatigue lives than IP for high strain ranges, due to early cracking and thus the life being dominated by crack propagation. Extrapolation of the curves show that this trend switches at lower strain ranges, a view shared by Pahlavanyali et al. [2] who performed tests on the same alloy over the same temperature range. A similar trend was found by Huang et al. [3] on M963 between 450 and 900 °C. Hyde et al. [4,5] carried out tests on the powder metallurgy nickel base superalloy, RR1000, between 300–650 °C and 300–750 °C, reporting that OOP generally gave longer lives than IP, owing to the transgranular nature of the OOP failure and the intergranular nature of the IP failure.

In light of these differences in fatigue lives found for various phase angles, it is clear that a greater understanding of failure mechanisms is required, especially in those cases where TMF lives are extremely short and most likely dominated by crack propagation. A ‘life to first crack’ approach has been utilised in modern lifing approaches, which involves taking a crack length reading at various inspection intervals, so it is essential that the TMF crack growth (TMFCG) behaviour is understood and the subsequent data is used for accurate lifing. Since the evolution of nickel disc alloys towards increased strength has also led to increased sensitivity to defects such as melt anomalies, or potential handling damage, a damage tolerance approach is critical.

The development of a TMFCG facility is experimentally challenging although a wide range of approaches have been attempted over recent decades. In particular the requirement for an appropriate combination of a crack monitoring method that couples effectively with the heating/cooling technique is a challenge. Induction heating has proven a popular choice for TMF since it allows for fast cycles to be achieved, it is easily accessible for all types of crack monitoring and is compatible for load and strain control testing since the coil design can be altered to accommodate an extensometer. In the 1970s and early 1980s, optical techniques were commonly utilised in conjunction with induction heating for crack monitoring [6,7,8], whilst the late 1980s and 1990s saw a shift towards direct current potential drop (DCPD) [9,10], where a current is applied to the specimen and probes detect the potential difference across the cracked surface as damage progresses. Compliance methods have also been used but have been found to give large scatter in results and variations in response upon loading due to a closure of crack faces behind the crack tip [11,12]. Optical methods provide a simple approach to crack monitoring, however the sensitivity is significantly reduced in comparison to DCPD methods, and also may be restricted by the heating apparatus being utilised. At a similar time, advancements such as quartz lamps allowed for alternative heating methods [13] with DCPD for load-controlled TMF. Furthermore, Jacobsson et al. [14,15] performed testing using resistance heating with an environmental scanning electron microscope (ESEM) under load control for IN718. The same authors also reported tests using an air heater and DCPD under strain control [16], showing quite clearly how the experimental methods for TMFCG contrast between authors due to opinions and merits of each method.

As stated, induction heating is a common choice since it provides rapid temperature cycles and can be used for complex geometries, however, the importance in coil design has been highlighted by Evans et al. [17] and the resultant thermal gradients have been explored by Beck et al. [18]. The same authors reported that care must be taken when combining induction with DCPD since eddy currents might interact with the current causing noise and interference. Resistance heating also allows for rapid temperature cycles but can cause localised heating of the crack tip [19], whilst air heaters minimise thermal gradients but give moderately slow heating and cooling rates. Finally, quartz lamps provide a uniform temperature distribution by cycling under such intense conditions, and relatively slowly, result in a decrease in bulb life [19]. It is evident that care must be taken when selecting the most effective combination of techniques down to the simplest detail.

The current work seeks to develop a test facility at Swansea University to enable TMFCG testing to be carried out accurately and effectively using the most suitable techniques. As such, induction coil heating has been considered with DCPD, along with each of their merits. A large aspect of this work therefore concentrated on the experimental development, ensuring compatibility, before moving onto isothermal baseline tests and finally TMFCG tests at various phase angles. An effective method of testing and analysing TMFCG of RR1000 will enhance understanding of the capability of the alloy, thus giving an efficient tool to predict lives of nickel-based superalloys for turbine disc applications.

## 2. Experimental Procedure

The material considered for the current investigation is fine grain RR1000, although the initial isothermal validation was carried out using Waspaloy, an earlier generation polycrystalline nickel base superalloy. The composition of these two alloys can be observed and compared in Table 1. RR1000 has a γ’ content of approximately 45% to ensure the required levels of strength are achieved, whilst a specific amount of chromium is present to control the amount of M_23_C_6_ that may form on grain boundaries [20]. Acceptable balance of tensile strength and crack growth resistance has been achieved through heat treatment, as such a near-solvus solution heat treatment, which is used in conjunction with air cooling [20].

The test machine used throughout this investigation was an Instron 1362 electric screw testing system, applying a 450 MPa maximum stress (11.25 kN load) at *R* = 0.1. A copper induction coil of approximately 6 mm diameter was wound four times to an external diameter of 43 mm to apply the heating with a fan cooler employed for forced air cooling, thus completing the thermal cycle between 300 and 700 °C in 80 s, providing a balanced linear heating/cooling rate of 10 °C/s. DCPD was then applied to monitor the crack growth, using an applied current of 15 amps.

**Figure 1:**
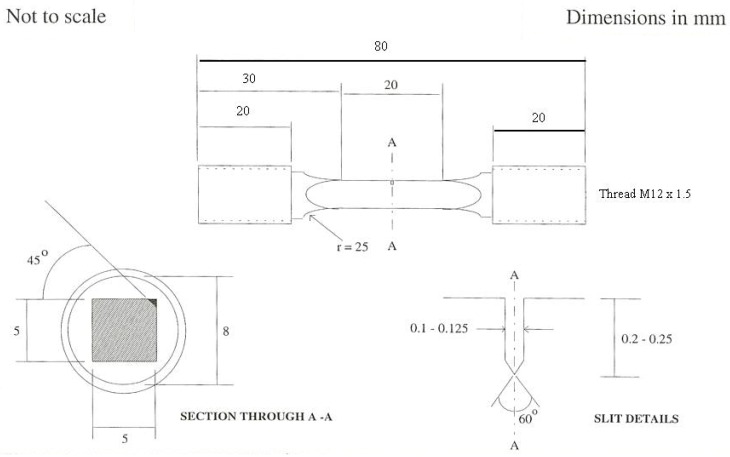
Corner crack specimen designed for thermo-mechanical fatigue (TMF) crack propagation in induction coil.

The Waspaloy specimens used for validation of the experimental set-up and thermal profiling were 10 × 10 mm and 7 × 7 mm square cross section specimens with a starter notch of 0.35 mm, manufactured using a mechanical cutting technique with a diamond edged saw, whilst the RR1000 specimens were smaller, 5 × 5 mm, with a starter notch of 0.22 mm, as shown in Figure 1. This small design was used to enable rapid thermal response resulting in cycles of the desired length.

The DCPD probe wires were spot welded above and below the notch as well as 5 mm above the notch in the centre of the adjacent face for referencing. Potential difference measurements were recorded by Dirlik Controls monitoring software from which crack growth rates could be calculated. Temperature was monitored by two sets of N-type thermocouples spot welded to the adjacent faces 2 mm below the notch plane, as will be described in the Thermal Profiling section. A PICO box provided rapid sampling of temperature for up to eight channels, which proved critical in ensuring that the thermal gradients were in a tolerable range. Since no standards or code of practice yet exist for TMFCG testing, acceptable conditions were derived from those set out in the *Strain Controlled TMF Code of Practice* [23], whereby a limit of +/−2% of the applied temperature range, Δ*T* or 10 °C was considered acceptable at maximum temperature, *T*_max_ (700 °C) and at minimum temperature, *T*_min_ (300 °C).

In order to avoid any residual effects from the machined starter notch and to ensure that the crack initiated from this location, a pre-cracking method was developed to improve repeatability. This involved the application of a sinusoidal waveform at 0.5 Hz, at room temperature and subsequently at the test temperature for isothermal tests or at the temperature at which the peak stress is experienced for TMF, for a measured amount of crack growth.

Isothermal testing was conducted using two different waveforms classified as fast and slow cycles. The former used a 4-s (1-1-1-1 trapezoidal) waveform whilst the latter used an 80-s (0.0125 Hz sinusoidal) waveform with the idea to allow comparison with the 80-s TMF cycles. The TMF tests were then carried out at 0° IP, 90° OOP and 180° OOP. In all cases the crack was grown as close to a final length of 2 mm as possible, that is, 40% of gauge width, before taking a replica, and then either unloaded in one piece or cycled to failure at room temperature. Post-test analysis could then be conducted on either the sectioned sample (cut at 45° to the sample surface for maximum crack size) or the exposed fracture surface as required. Analysis was conducted on either a JSM 6100 Scanning Electron Microscope (SEM) (JEOL, Tokyo, Japan) or Philips XL30 SEM equipped with Electron Back Scatter Diffraction (EBSD) to determine the failure mechanisms for each condition.

## 3. Results and Discussion

### 3.1. Preliminary Waspaloy Isothermal Tests

**Figure 2:**
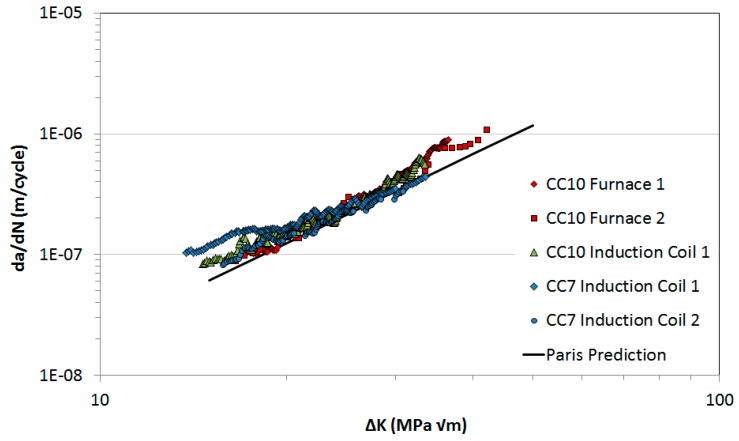
Waspaloy crack growth rates vs. stress intensity range: furnace and induction coil comparisons at 650 °C, 450 MPa and R = 0.1.

As previously discussed, a significant challenge in TMFCG is finding a compatible combination of heating and crack monitoring techniques. Therefore, the first step of experimental work consisted of carrying out isothermal fatigue (IF) tests at 650 °C, 550 MPa and *R* = 0.1 with a 1-1-1-1 trapezoidal waveform. Waspaloy specimens with a 10 × 10 mm, CC10 (Corner Crack, 10 mm gauge width), cross-section were tested in a conventional split radiant furnace, and compared to tests where heating was provided through the use of an induction coil. Requirements of the testing in an induction coil were twofold, firstly to determine whether the induction coil produced repeatable results and secondly that the results were comparable to tests undertaken in the radiant furnace. The results of the preliminary tests are shown in Figure 2 and it can be seen that little variation occurs between the induction coil tests and also that these results are fully consistent with furnace results for the 10 × 10-mm set-up. Since the TMFCG tests were scheduled to be conducted on reduced size 5 × 5 mm cross-section, a smaller 7 × 7-mm CC7 (Corner Crack, 7-mm gauge width) Waspaloy specimen was tested under the same conditions to confirm the scale invariance of growth rates in specimens of these sizes. Fully consistent results can be observed in Figure 2, providing confidence in the experimental set-up provided. Also provided is a Paris law prediction based on a crack growth model.

The Paris law has been used historically to predict crack growth rates at various stress intensities, using the theory that as the number of cycles increases the crack growth rate increases thus the change in crack length must be measured with respect to the change in cycles [24],

$$\frac{da}{dN}=C\Delta K^{m}$$

where *a* is the crack length, *N* is the number of cycles, *C* and *m* are material constants and ∆*K* is the stress intensity range (stress field at the crack tip), which can be calculated by,

$$\Delta K=\Delta \sigma Y\sqrt{\pi a}$$

where ∆σ is the range of stress and Y is a dimensionless geometry parameter.

### 3.2. Thermal Profiling

With confidence developed in the experimental set-up for isothermal crack growth measurements, the next step was the introduction of thermal cycling. This was performed independently of the DCPD detection methods at first in order to simplify the procedure.

**Figure 3:**
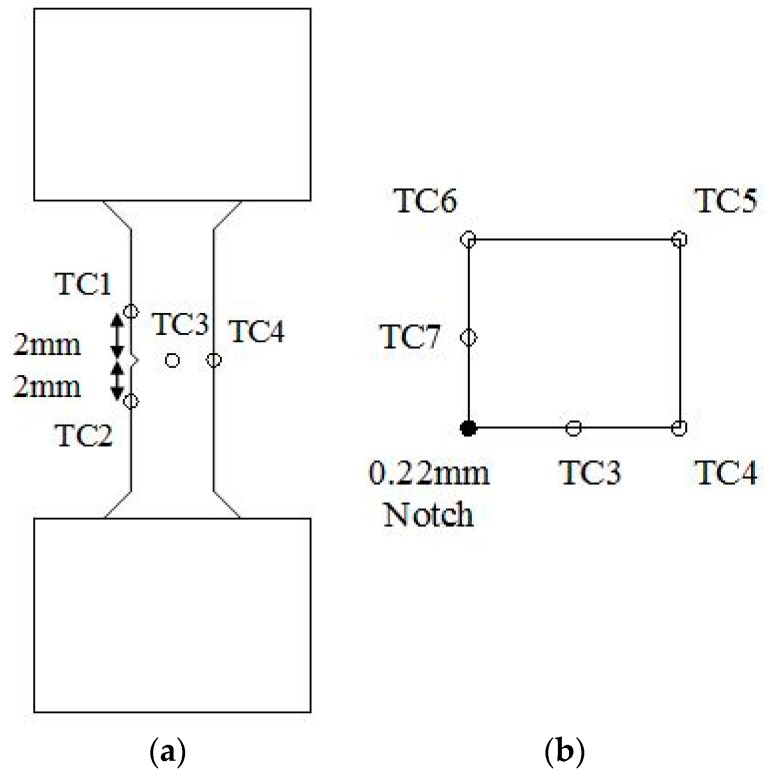
Thermocouple locations for thermal profiling (**a**) side view and (**b**) plan view.

Seven locations were selected for thermocouples, similar to those presented by Jacques et al. [25], to monitor the thermal profiles across the specimen. Figure 3 shows the locations used for the current work, showing five thermocouples around the notch plane on the faces and corners, with a set 2 mm above the plane at the notch and a set 2 mm below. This therefore gave a full representation of the response of the heating and cooling from induction heating the specimen axially, ensuring thermal gradients were minimised and uniform thermal cycling was achieved.

**Figure 4:**
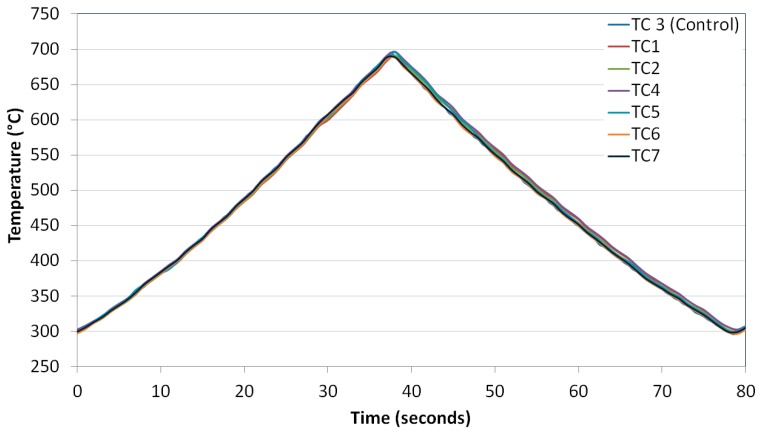
Thermocouple responses during the 80 s cycle.

The cycle targeted was 40 s heat and 40 s cool, giving a total cycle time of 80 s. The coil and fan cooler were positioned until this desired response was achieved in line with the TMF code of practice [23]. Although the guidelines set out were designed for strain controlled TMF experiments, since no relevant standard or code of practice (CoP) has yet been developed for TMF crack growth testing, it was decided that these guidelines were the most appropriate. The CoP demands that for induction heating that “the axial temperature gradients within the gauge length should not exceed ±10 °C or ±2% of the temperature range (°C)”. Given that the temperature range of 400 °C was utilised in the current experiments, it was clear that the ±2% Δ*T* gave more accurate readings of ±8 °C, so this was the guideline followed. The response of the thermocouples and the uniformity of the cycle achieved are presented in Figure 4.

**Figure 5:**
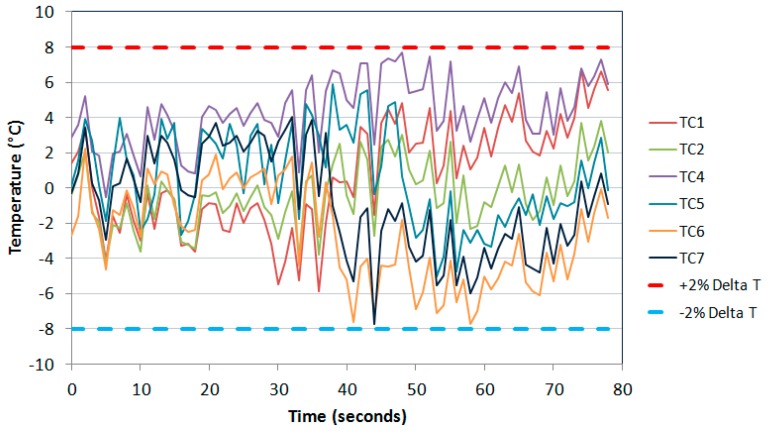
Thermocouple deviation over the 80-s thermal cycle.

The deviation with respect to TC3 can be seen in Figure 5. It is evident that the experimental set-up resulted in a tolerable thermal response sitting within the bounds set out by the CoP, so repeatability of this set-up was imperative. The rig was dismantled in order to replicate a specimen change-over or a rig calibration and returned to the recorded positions giving rise to a similar satisfactory response.

### 3.3. RR1000 Isothermal Testing

Before TMFCG testing, isothermal fatigue tests were undertaken on the 5 × 5 mm RR1000 specimens to provide baseline data and thus allow direct comparisons with the respective TMFCG tests. These isothermal tests were selected to correspond to the temperature at which the TMF test was exposed to the maximum stress, thus the most damaging part of the cycle (300 °C for 180° OOP, 500 °C for 90° OOP and 700 °C for IP).

**Figure 6:**
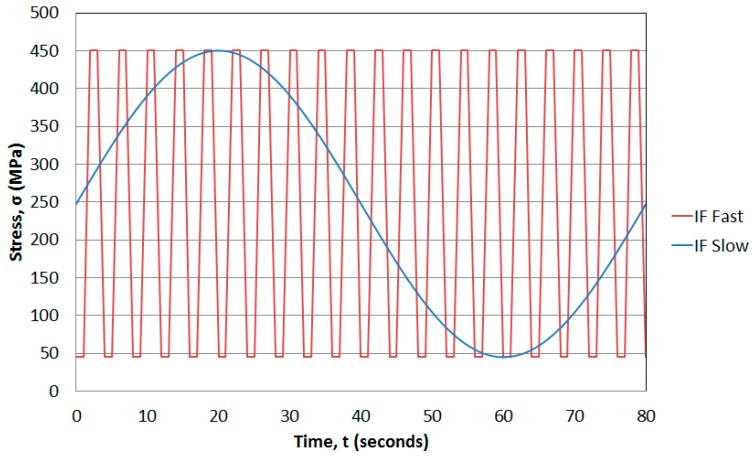
Stress response for ‘fast’ and ‘slow’ isothermal fatigue (IF) tests.

**Figure 7:**
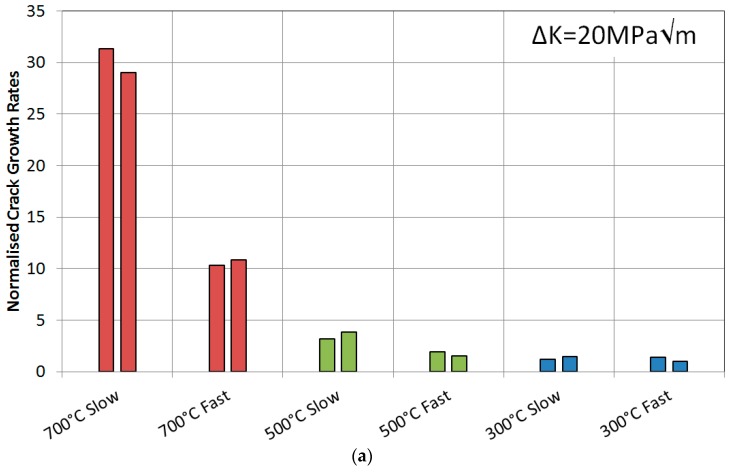

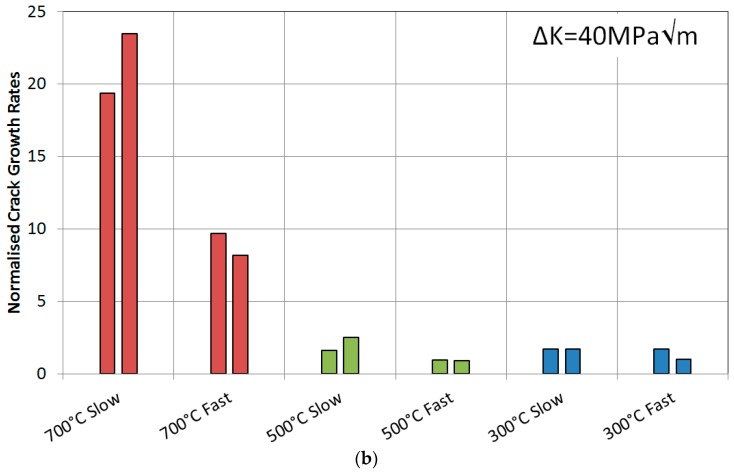
Normalised crack growth rates at a stress intensity range of (**a**) 20 MPa√m and (**b**) 40 MPa√m for RR1000 under isothermal conditions.

Two types of isothermal tests were used in terms of loading, a fast 1-1-1-1 (4 s) trapezoidal and a slow 0.0125 Hz (80 s) sinusoidal waveform, in order to observe the relevance of the cycle length on the TMF tests, as presented in Figure 6. Two tests were then carried out for each waveform at each temperature, giving 12 results in total. The responses can be seen in Figure 7, showing that the higher the temperature the faster the crack growth rates, as expected due to the introduction of oxidation and with some creep effects at these higher temperatures, in accordance with work by Tong et al. [26]. These plots have been normalised by the slowest crack growth rate achieved in the 300 °C fast IF test for the respective ∆*K* value.

Each temperature can then be scrutinised by cycle type. Taking the 700 °C tests, the slow cycle resulted in accelerated growth when compared with the fast cycle. This is due to the specimen spending a longer time in the high temperature/high stress regime i.e., the slow cycle spends longer at the turning point of the peak stress region. This presumably allows for significant oxidation at the crack tip promoting more rapid growth. The 500 °C tests showed a similar response in that the slow cycle gave rise to faster growth rates than the fast cycle. However this time the difference is less severe, although it is apparent that the temperature is still high enough to allow time dependent mechanisms to provide acceleration in the growth rates. Finally, the 300 °C data suggests that these damage mechanisms do not operate at such low temperatures since there is no difference between the two rates of the cycles.

**Figure 8:**
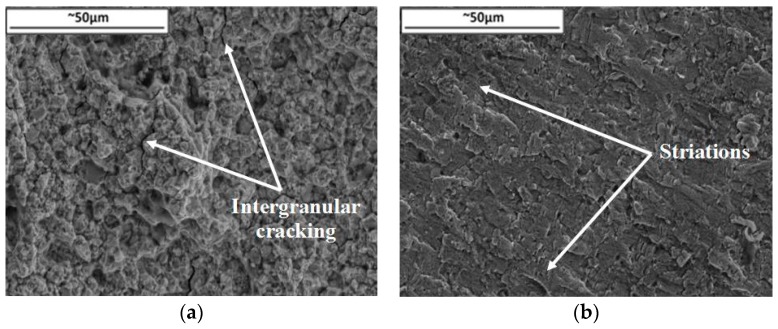
Fractography of slow cycles at ∆*K* 20 MPa√m for (**a**) 700 °C and (**b**) 300 °C.

Figure 8 provides fractographs for slow cycles undertaken at (a) 700 °C and (b) 300 °C, which is consistent with the data provided in the TMFCG experiments in that the high temperature isothermal slow cycles gave rise to intergranular dominant fracture. This supports the theory that oxidation and potentially creep are playing a significant role by weakening the grain boundaries, providing an easy path for the crack to follow. The lower temperature of 300 °C sees a complete change in the damage mechanism, this time giving rise to a transgranular dominant failure, as can be seen in Figure 8b, with striations now more visible than at 700 °C as the effect of oxidation and creep is reduced.

### 3.4. TMFCG Testing

**Figure 9:**
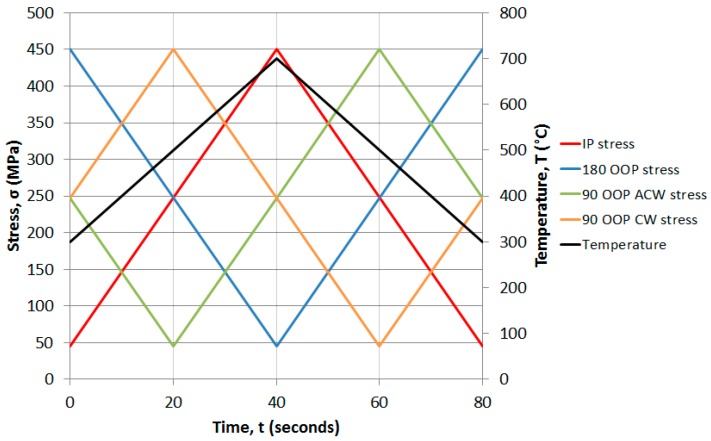
Stress cycles vs. temperature for all TMF conditions. IP: in-phase; OOP: out-of-phase; CW: clockwise; ACW: anticlockwise.

**Figure 10:**
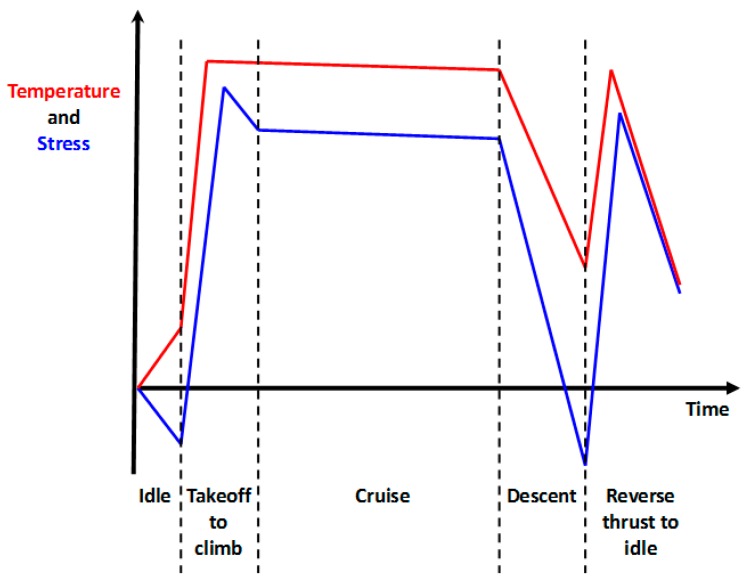
Turbine conditions during a civil engine flight cycle based on Marchand et al. [27].

The progress in thermal profiling and isothermal testing allowed the work to concentrate on the main goal of the investigation, carrying out TMFCG experiments on RR1000. The thermal profile was combined with the loading profile through the Dirlik Control software to thus allow crack monitoring under TMF conditions. Key phase angles desired were IP, 90° OOP and 180° OOP (Figure 9) in order to determine the effects of different stages of a typical flight cycle, as presented Figure 10.

**Figure 11:**
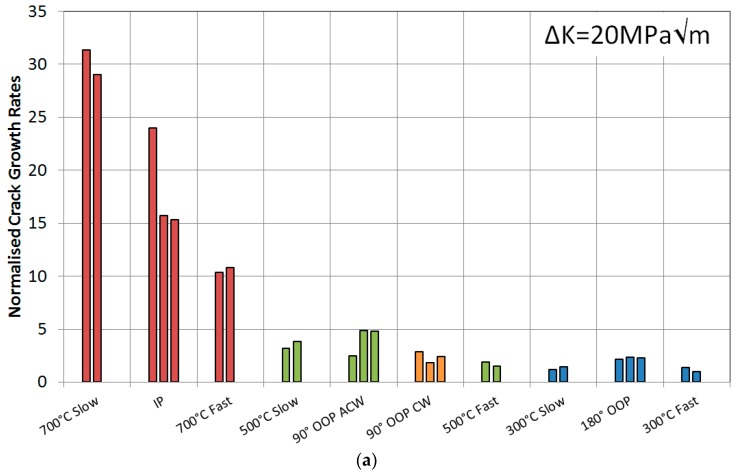

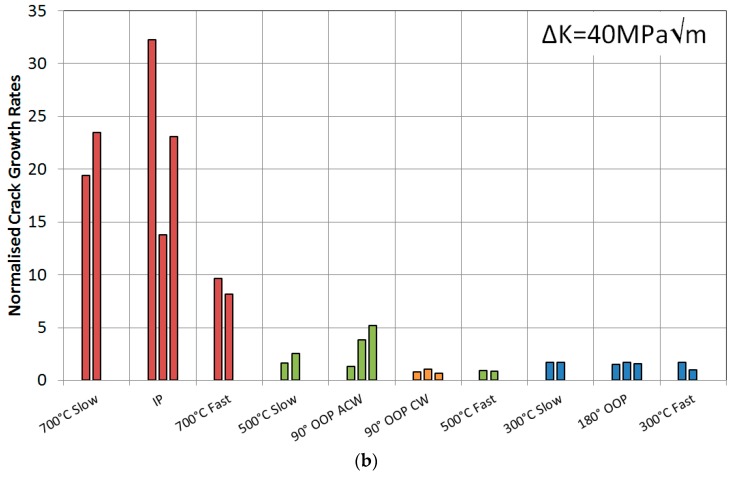
Normalised crack growth rates at a stress intensity range of (**a**) 20 MPa√m and (**b**) 40 MPa√m for RR1000 under TMF and isothermal conditions.

The first set of data was collected for IP tests which were compared directly to the 700 °C isothermal results, shown in Figure 11. First of all, the repeatability of the three IP tests gave confidence that the experimental technique was working, particularly for this phase angle. Slower crack growth rates were observed for IP when compared to the slow 700 °C IF cycle, which was expected since the IF test spent the entirety at 700 °C resulting in a longer time in the high temperature/high stress regime, thus promoting increased creep and oxidation damage. The fast IF test spent less time than both of these cycle types in this regime thus giving rise to a slower crack growth rate than both.

Figure 11 also presents the 500 °C IF data plotted along with the 90° OOP data in two directions, clockwise and anti-clockwise. A similar response to the 700 °C IF was found in 500 °C IF in that the slower cycle gave rise to a faster growth rate as was discussed earlier, but the TMF results provided interesting results. The anticlockwise cycle (ACW) caused a slightly faster crack growth rate than clockwise (CW) suggesting that there is a damage mechanism interaction difference when the specimen is loaded ‘hot’ and ‘cool’, and this is discussed subsequently in conjunction with the fractography results. The 180° OOP shows a faster crack growth rate than the 300 °C IF tests, suggesting that time spent at high temperature promoted a faster growth rate either through relaxation of compressive crack tip stresses or enhanced oxidation.

**Figure 12:**
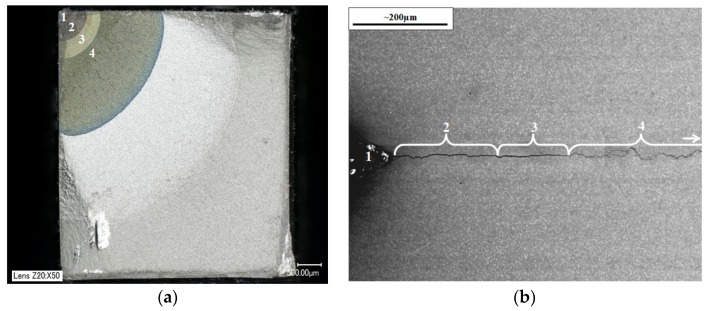
Notch, pre-cracking and test recognition from the two types of post-test analysis, (**a**) specimen stressed to failure and (**b**) unloaded for sectioning.

The resultant fracture surfaces were analysed to establish the behaviour and failure mechanisms present in each case. Figure 12 shows the two types of post-test analysis methods used, where each stage was identified through oxidation colour (Figure 12a) and change in crack mechanism (Figure 12b). The corresponding numbers stand for:

1. Pre-machined notch region;
2. Room temperature pre-cracking;
3. Test temperature (IF) or temperature at which maximum stress is experienced (TMF) pre-cracking;
4. IF or TMF test where the crack is grown to about 2 mm.

**Figure 13:**
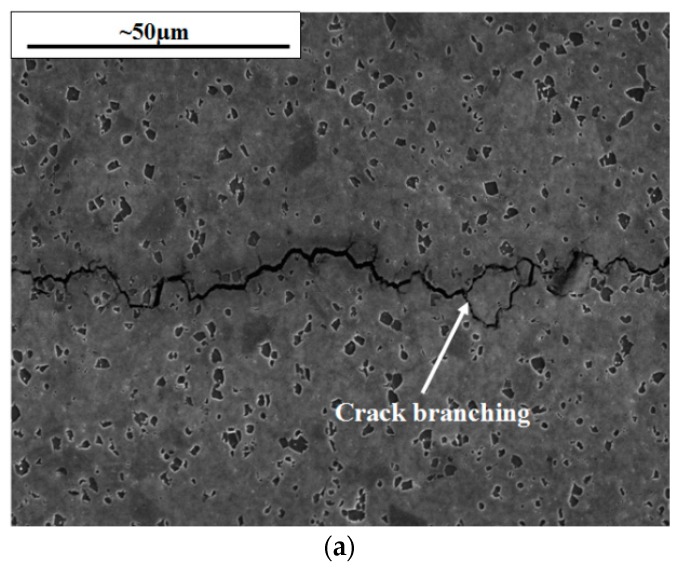

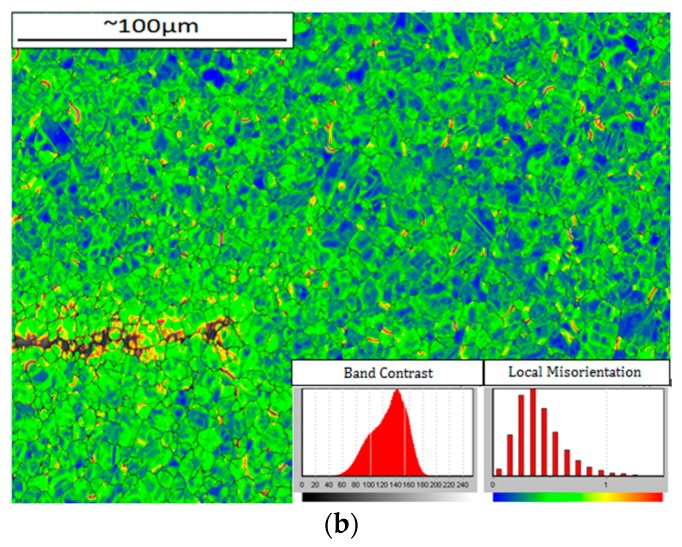
Intergranular cracking from IP testing shown by (**a**) an SEM image presenting crack branching and (**b**) an EBSD image of the crack tip.

Fractography was therefore carried out in region 4, to compare the different test conditions. As the IF 700 °C fractography suggested, the IP conditions gave rise to intergranular dominated failure as a result of the time dependent effects at the high temperature/high stress regime. This is confirmed in Figure 13, where (a) also shows evidence of crack branching, in some cases rejoining the main crack, such as the example shown, whilst in others the crack branch halts giving an irregular crack path. This has been documented in previous literature to be a result of the crack approaching two simultaneous slip bands causing their activation due to the crack tip being highly stressed [15,21]. The EBSD study in (b) shows the crack tip at the end of the test, so the specimen has been unloaded, however, it does still show some local misorientation of grains, relative to neighbouring pixels, spanning two grains above, below and ahead of the crack tip.

**Figure 14:**
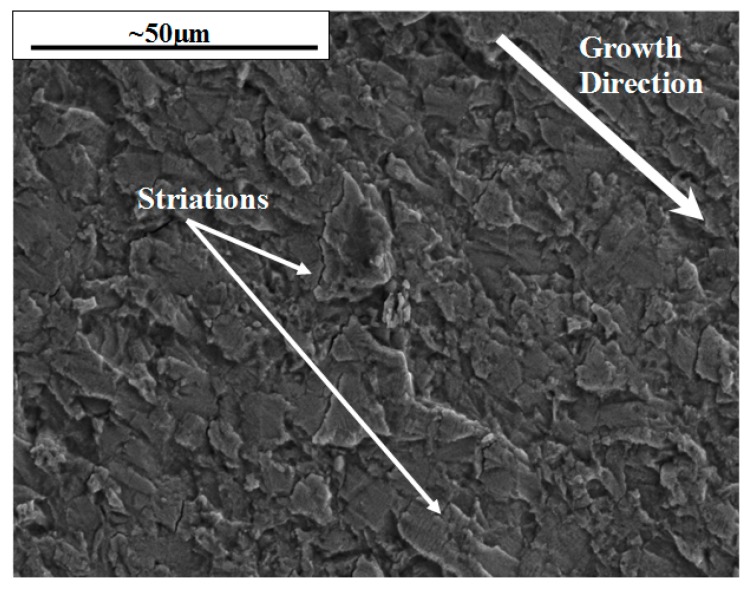
Transgranular dominant failure for 180° OOP tests showing the fracture surface at ∆*K* = 20 MPa√m.

When IP is compared to 180° OOP a considerable change in damage mechanism arises. A transgranular dominant crack is observed under these conditions with a large number of striations present within the microstructure, shown in Figure 14a. The distance between these striations were measured and found to increase significantly from start to finish from about 0.1 to 2 µm as the crack progressed at an increasing pace.

These IP and 180° OOP fracture surfaces agree with the histograms plotted in Figure 11 where it is clear that IP leads to faster growth rates than OOP. This is therefore a result of the change in dominant failure mechanism from IP to OOP as intergranular to transgranular. This concurs with findings from Hyde et al. [4,5] on the same alloy over a similar temperature range, 300–650 °C and 300–750 °C, and gives similar results to those produced by Jacobsson et al. [14] with Inconel 718.

**Figure 15:**
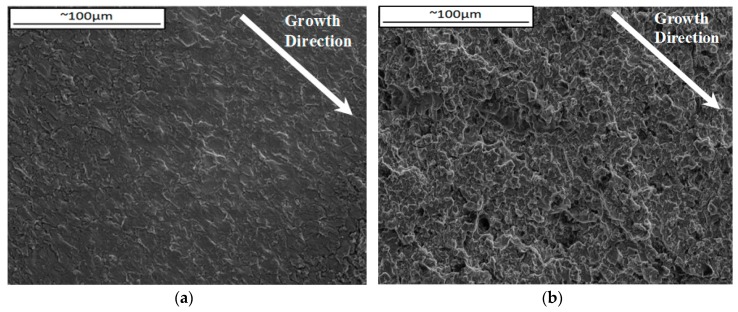
Fracture surfaces of 90° OOP at ∆*K* = 20 MPa√m test in (**a**) ACW and (**b**) CW loading directions.

To the knowledge of the authors very little work has been carried out on diamond phase angles, in fact Pahlavanyali et al. [2,22,28] and Yedra et al. [29] seem to be the only authors who have documented these cycles under standard TMF conditions. This has resulted in very limited data being available to compare any experimental results under such conditions, so the 90° OOP conclusions have been drawn merely from observations and theories from the current work. In general, the plots in Figure 11 presented slightly faster crack growth rates in the ACW cycle compared to the CW cycle, whose respective fractography can be observed in Figure 15.

**Figure 16:**
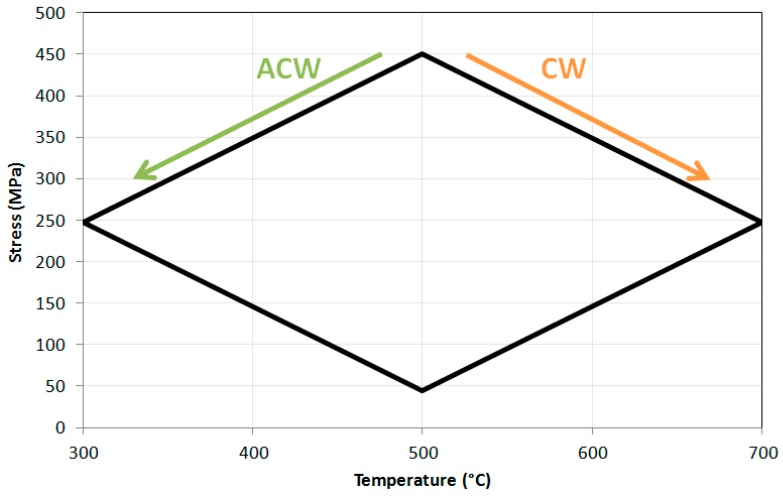
Stress plotted against temperature for 90° OOP cycles in the ACW and CW loading directions.

According to these micrographs it appears that ACW loading direction caused transgranular failure whilst CW resulted in more mixed mode failure. This loading direction has been described in Figure 16.

Starting from the top of the plot in Figure 16 (maximum stress, mean temperature) and following it in the clockwise direction, it is clear that the specimen is being unloaded at high temperatures, and since it remains in tension throughout the test (*R* = 0.1) there is minimal closure of the crack. With a dormant crack and high temperatures, oxidation occurs at the crack tip, damaging/oxidising the grain boundaries a small distance ahead of the tip. As tensile re-loading occurs in the cycle, this takes place at low temperatures so even though little to no oxidation occurs here, the grain boundaries have already been degraded by the unloading (high temperature) stage, thus giving rise to a relatively slow intergranular mixed mode fracture. Li et al. describes that oxides form at the crack tip and it is the rupture of these oxides that cause the environmentally-assisted crack growth [30].

In the ACW cycle the specimen now unloads at low temperatures, therefore a smaller damaged zone due to oxidation is obtained. The loading part of the cycle happens at higher temperatures so oxidation does take place, however, since the cycle is so fast dynamic, transgranular failure prevails as the crack progresses faster than the oxidation process, since fresh material is constantly being exposed.

**Figure 17:**
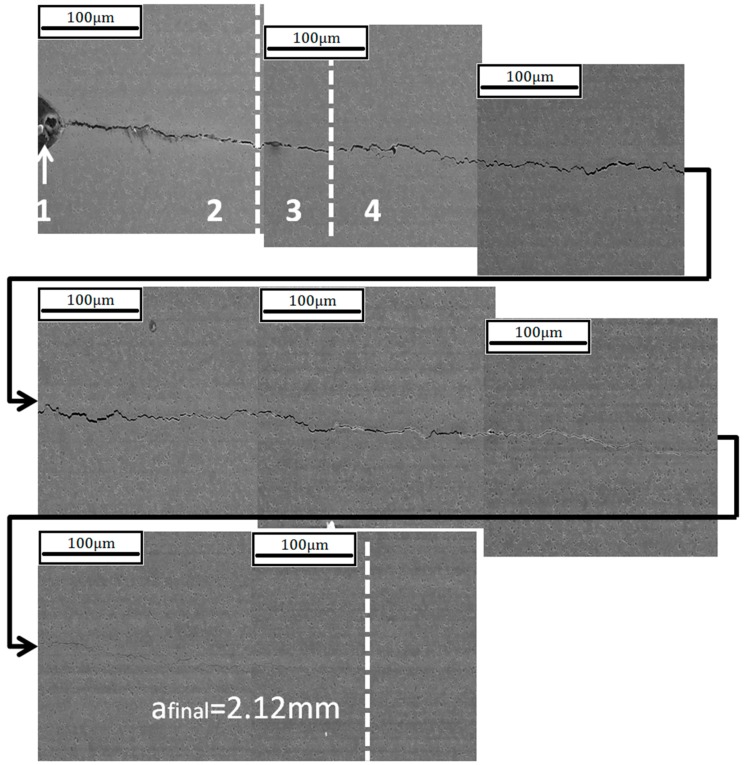
Crack progression of the 80 s 90° OOP ACW test.

**Figure 18:**
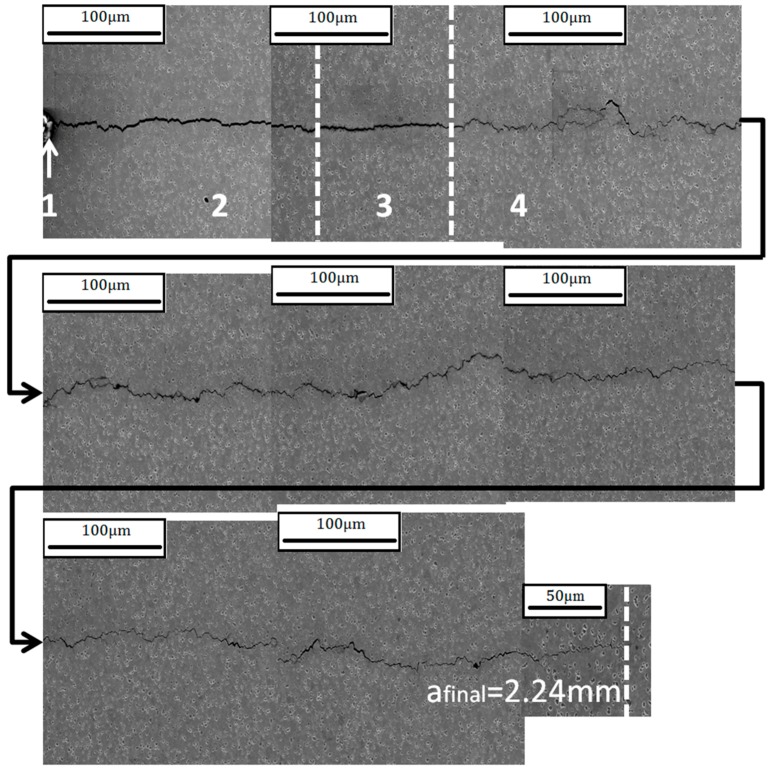
Crack progression of the 500 s 90° OOP ACW test.

To test this theory a further ACW test was carried out. However, instead of the standard 80-s cycle, a 500-s cycle was used in order to attempt to manipulate the damage mechanism. This was to investigate if the oxidation process would overtake the crack growth process causing damage ahead of the crack tip resulting in an intergranular failure. Figure 17 shows the crack progression of the faster 80-s cycle whilst Figure 18 presents the slower 500-s cycle, with the four respective stages of the test highlighted the same as in Figure 12. The pre-cracking contributions to the crack growth are illustrated along with the damage caused by the TMF test through to the crack tip. It is clear that the 500-s cycle gave rise to a more intergranular dominated failure than the 80-s cycle, suggesting that the increased exposure time to the higher temperature upon loading allowed for the oxygen to penetrate beyond the crack tip damaging the grain boundaries.

**Figure 19:**
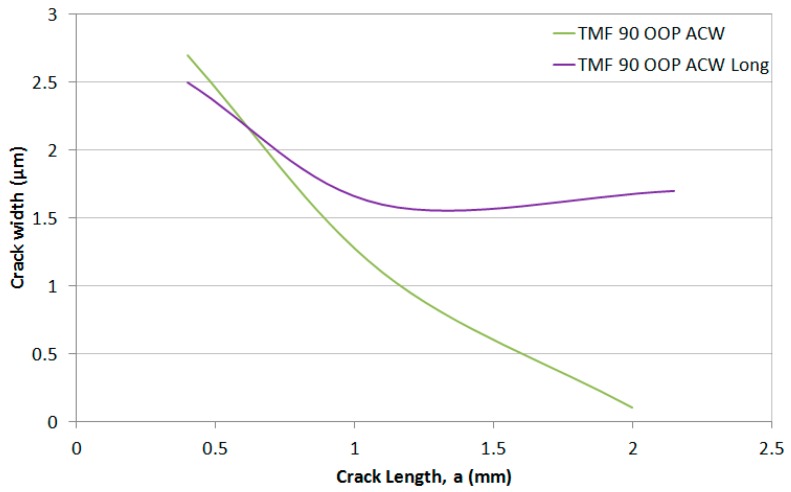
Crack width plotted against crack length for both 90° OOP ACW tests during stage 4 of the crack (TMF test).

**Figure 20:**
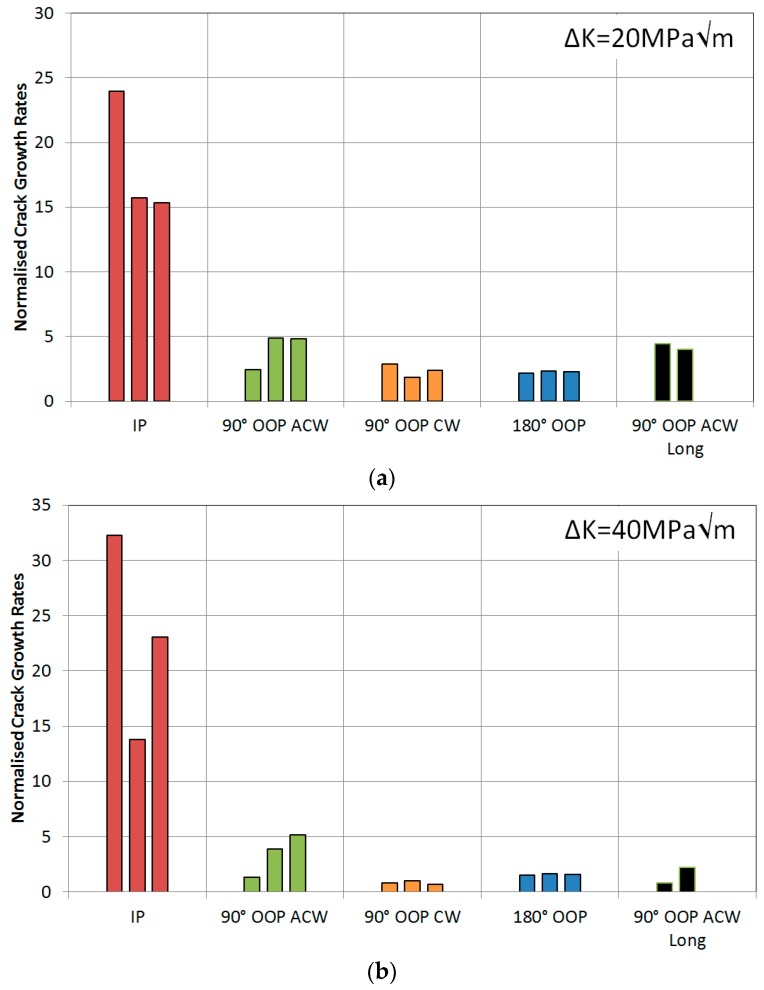
Normalised crack growth rates at a stress intensity range of (**a**) 20 MPa√m and (**b**) 40 MPa√m for RR1000 under TMF conditions.

It can also be observed that the 500-s cycle resulted in a wider crack throughout the test, implying that the increased amount of oxides forming at the crack tip created a wedge between the top and bottom surfaces of the crack, resulting in crack tip blunting. This width difference, measured perpendicularly to the crack flanks, has been shown graphically in Figure 19, confirming the wider crack from the 500-s cycle. This crack blunting theory is supported by the data appearance on the histogram in Figure 20 and the number of cycles to critical crack size in Table 2. The low values of this 500-s cycle test, particularly at high stress intensities, means that the growth rate is almost independent from the stress intensity since it would be expected that this longer cycle would result in a faster crack growth rate due to creep and oxidation effects causing the intergranular failure.

**Figure 21:**
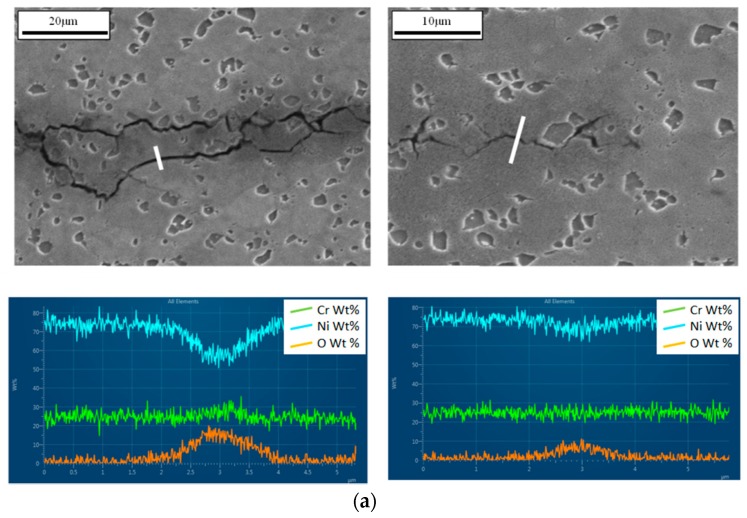

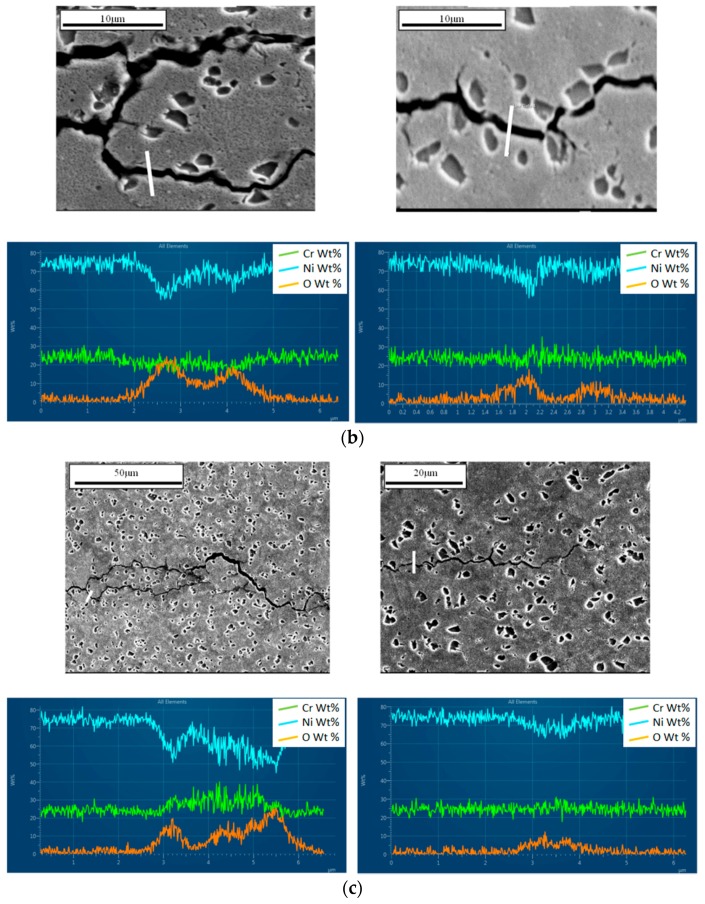
Energy dispersive X-ray spectroscopy (EDX) line scans taken near the start of the test and near the crack tip for (**a**) ACW 80-s cycle; (**b**) CW 80-s cycle and (**c**) ACW 500-s cycle.

In order to confirm why crack tip blunting was occurring, energy dispersive X-ray spectroscopy (EDX) was carried out across the crack. In order to attain such micrographs, the tests were terminated at 2.2 mm, before crack growth accelerated to failure, and sectioned at 45° to the surface, where maximum crack length is recorded, using a cutting wheel. The areas analysed were at a = 0.8 mm (near the beginning of the test where a slow moving crack is present) and at a = 2.15 mm (near the crack tip where a fast moving crack is present), as shown in Figure 21.

The first observation is that oxygen (orange data) ingress is occurring as expected with a larger concentration at a position near the start of the crack where exposure times are higher. Secondly, it is clear that the oxidation levels are much higher in the 80-s CW test than the 80-s ACW test. Meanwhile the oxygen levels for the 500-s ACW test exceeds both. It must be considered that EDX is not accurate for elements with small atomic numbers, therefore this analysis is not definitive but provides the inclination that oxygen has caused variation in failure mechanisms with a transition to intergranular crack growth.

In terms of crack tip blunting, the width of the cracks suggests that an increased amount of oxygen in the latter two tests, Figure 21b,c, gave rise to the blunting and thus retarding the crack growth rates due to the decrease in stress concentration at the tip. This is supported by the relatively low values on the histogram in Figure 20.

## 4. Conclusions

A TMFCG method and rig has been developed at Swansea University to carry out tests exploring the TMFCG behaviour of the nickel base superalloy, RR1000, aiding the lifing of turbine discs. The tests have proven to be promising, enabling the authors to draw a number of conclusions.

- Induction heating appears to have no effect in terms of crack tip heating or interference with DCPD crack monitoring techniques, and the combination of the two therefore appears satisfactory both for IF crack growth and TMFCG load control tests.
- Time dependence plays a significant role when comparing ‘fast’ and ‘slow’ cycle IF tests at temperatures higher than 500 °C.
- IP conditions result in faster crack growth rates than OOP, due to the high stress and high temperature regime being more damaging in terms of creep and oxidation, giving rise to intergranular failure compared to the more transgranular-dominated failure with OOP.
- Diamond cycles are sensitive to loading direction, as shown by the 90° OOP, with slightly increased growth rates resulting from the ACW cycles.
- A theory has been tested to determine if oxidation rates are responsible for the change in damage mechanism between the two. Early signs suggest that this theory holds such that a fast moving crack at high temperatures oxidises less than a dormant crack in the same temperature range (right hand side of Figure 16) which results in transgranular failure in the former and a more brittle intergranular dominated failure in the latter.
- Oxygen films have been found to build up on the upper and lower surfaces of the crack, particularly during the highly oxidising CW and 500 s ACW cycles, resulting in crack tip blunting and thus retarded crack growth rates.
- Further work is required to confirm the effect of oxygen under these test conditions. Vacuum tests and dwell tests would be useful to identify and confirm if oxidation is the main factor involved in the intergranular aspect of failure in the diamond cycles.

## Figures and Tables

**Table 1:** Waspaloy and RR1000 compositions (wt %) [21,22].

Alloy	Ni	Cr	Co	Mo	Ta	Al	Ti	Hf	C	B	Zr
Waspaloy	bal	19.5	13.5	4.3	–	1.3	3.0	–	0.08	0.006	–
RR1000	bal	15.0	18.5	5.0	2.0	3.0	3.6	0.5	0.03	0.02	0.06

**Table 2:** Summary of number of cycles to reach 2.2 mm for each test condition.

Isothermal/TMF	Cycle	Mean No. of Cycles to 2 mm	Mean Time to 2 mm (Hours)
IF	700 °C Slow	1180	26
	700 °C Fast	2450	3
	500 °C Slow	8700	193
	500 °C Fast	19,500	22
	300 °C Slow	36,000	800
	300 °C Fast	45,000	50
TMF	IP	2800	62
	90° OOP CW	9300	207
	90° OOP ACW	8000	178
	90° OOP ACW Long	6700	931
	180° OOP	14,600	324
